# Assessment of Choroidal Thickness in Healthy and Glaucomatous Eyes Using Swept Source Optical Coherence Tomography

**DOI:** 10.1371/journal.pone.0109683

**Published:** 2014-10-08

**Authors:** Chunwei Zhang, Andrew J. Tatham, Felipe A. Medeiros, Linda M. Zangwill, Zhiyong Yang, Robert N. Weinreb

**Affiliations:** 1 Hamilton Glaucoma Center and Department of Ophthalmology, University of California San Diego, San Diego, California, United States of America; 2 Department of Ophthalmology, the First Affiliated Hospital, Harbin Medical University, Harbin, China; Medical University Graz, Austria

## Abstract

**Purpose:**

To evaluate choroidal thickness (CT) in healthy and glaucomatous eyes using Swept Source Optical Coherence Tomography (SS-OCT).

**Methods:**

A cross-sectional observational study of 216 eyes of 140 subjects with glaucoma and 106 eyes of 67 healthy subjects enrolled in the Diagnostic Innovations in Glaucoma Study. CT was assessed from wide-field (12×9 mm) SS-OCT scans. The association between CT and potential confounding variables including age, gender, axial length, intraocular pressure, central corneal thickness and ocular perfusion pressure was examined using univariable and multivariable regression analyses.

**Results:**

Overall CT was thinner in glaucomatous eyes with a mean (± standard deviation) of 157.7±48.5 µm in glaucoma compared to 179.9±36.1 µm in healthy eyes (P<0.001). The choroid was thinner in both the peripapillary and macular regions in glaucoma compared to controls. Mean peripapillary CT was 154.1±44.1 µm and 134.0±56.9 µm (P<0.001) and macular CT 199.3±46.1 µm and 176.2±57.5 µm (P<0.001) for healthy and glaucomatous eyes respectively. However, older age (P<0.001) and longer axial length (P<0.001) were also associated with thinner choroid and when differences in age and axial length between glaucomatous and healthy subjects were accounted for, glaucoma was not significantly associated with CT. There was also no association between glaucoma severity and CT.

**Conclusions:**

Glaucoma was not associated with CT measured using SS-OCT; however, older age and longer axial length were associated with thinner choroid so should be considered when interpreting CT measurements.

## Introduction

Glaucoma is characterized by progressive damage to retinal ganglion cells leading to optic nerve head (ONH) morphological changes, thinning of the retinal nerve fiber layer and loss of visual field [1]. The underlying mechanism of damage is incompletely understood, but is likely multifactorial [2]. Raised intraocular pressure (IOP) is a major risk factor [3]–[6]. It also has been proposed that the microcirculation and vascular perfusion can contribute to the health of the optic nerve. As choroidal derived vasculature perfuses the prelaminar area of the ONH [7], there is interest in the role of the choroid in the pathogenesis of glaucoma [8].

Initial histological studies supported an association between choroidal morphology and glaucoma, with several reports of thinner choroid in glaucomatous compared to non-glaucomatous eyes [9]–[11]. Reduced thickness was thought to be due to loss of the innermost choroidal vasculature and it was suggested that choroidal thinning might be associated with choroidal insufficiency which could contribute to glaucomatous retinal ganglion cell damage. However, further evidence is needed to support or refute this theory, particularly as histological studies have limitations due to fixation methods and delays between death and fixation that may induce artifacts. Also, previous histological studies were limited to small numbers of patients and did not control for other factors now known to influence choroidal thickness (CT) including age and axial length [9], [12], [13].

It is now possible to obtain in vivo images of the choroid using enhanced depth imaging optical coherence tomography (EDI-OCT), a modified version of spectral domain OCT (SD-OCT) [14]. Enhanced depth imaging has been used to examine macular CT in healthy and glaucomatous eyes, with most studies finding no significant difference [15]–[18]. However, although enhanced depth imaging allows visualization of the choroid, the choroidal-scleral boundary may be difficult to discern. Furthermore, as choroidal segmentation software is not readily available, the assessment of CT has often relied on manual measurements at localized points [19]–[20]. Recently, a new generation of high-penetration OCT devices has been introduced with the potential to improve assessment of the choroid [21]. These Fourier-domain OCT devices are based on an alternative approach to image acquisition, known as Swept Source OCT (SS-OCT). SS-OCT devices use a tunable laser (i.e., one whose wavelength of operation can be altered in a controlled manner) and photodetectors instead of the silicone-based, line-scan, charge-coupled device camera used in SD-OCT systems. These innovations reduce light scatter by the retinal pigment epithelium and therefore enable better visualization of deeper ocular structures including the choroid. Previous studies have shown the potential of using SS-OCT for choroidal imaging [22] especially given the availability of software for segmentation of multiple retinal layers and the choroid [23]. SS-OCT also provides the capability of a wide field 12×9 mm scan enabling simultaneous imaging of the macula and ONH and measurement of CT over a larger area than previously possible in a single scan. Peripapillary and macular CT can therefore be calculated from a single scan.

The aim of the present study was to use SS-OCT to assess CT in healthy and glaucomatous eyes. We also assessed the effect of factors including age, gender, race, axial length, retinal nerve fiber layer (RNFL) thickness, mean deviation (MD), intraocular pressure (IOP), blood pressure (BP) and ocular perfusion pressure (OPP) on choroidal thickness.

## Methods

This was a cross-sectional observational study of participants from the Diagnostic Innovations in Glaucoma Study (DIGS) at the University of California San Diego (UCSD). The DIGS is a prospective longitudinal study designed to evaluate optic nerve structure and visual function in glaucoma. Informed consent was obtained from all participants, and the institutional review board and human subjects committee at UCSD prospectively approved all methods. All study methods adhered to the tenets of the Declaration of Helsinki for research involving human subjects and the study was conducted in accordance with the regulations of the Health Insurance Portability and Accountability Act.

Methodological details have been described in detail previously [24]. At each visit during follow-up, subjects underwent a comprehensive ophthalmologic examination including review of medical history, BP, best-corrected visual acuity, slit-lamp biomicroscopy, IOP measurement, gonioscopy, dilated fundoscopic examination, stereoscopic optic disc photography, and standard automated perimetry (SAP) using the Swedish Interactive Threshold Algorithm (Standard 24-2). Central corneal thickness (CCT) was measured with ultrasound Pachymetry (DGH Technology Inc, Exton, PA) and axial length measurement using the IOL Master (Carl Zeiss Meditec, Dublin, CA) was also performed. Diastolic and systolic ocular perfusion pressure was calculated as diastolic or systolic BP minus IOP respectively. Objective measurements of choroidal and RNFL thickness were obtained using the swept source Deep Range Imaging OCT (DRI-OCT-1 Atlantis, Topcon, Inc., Tokyo, Japan). The study included only subjects with open angles on gonioscopy. Subjects were excluded if they presented with a best-corrected visual acuity less than 20/40, spherical refraction outside ±5.0 diopters or cylinder correction outside 3.0 diopters, or any other ocular or systemic disease that could affect the optic nerve or visual field.

The study included 322 eyes of 207 participants, including 106 healthy and 216 glaucomatous eyes. Eyes were classified as glaucoma if they had repeatable (≥2 consecutive) abnormal SAP test results or progressive glaucomatous changes on masked grading of optic disc stereophotographs. Healthy subjects were recruited from the general population through advertisements and from the staff and employees of the University of California San Diego. Healthy eyes had IOP≤21 mmHg, with no history of increased IOP and a normal SAP result.

### Standard Automated Perimetry

SAP was performed using the Humphrey Field Analyzer II (Carl Zeiss Meditec, Dublin, CA, USA) and the 24-2 Swedish interactive threshold algorithm (SITA Standard 24-2, Carl Zeiss Meditec, Inc., Dublin, CA, USA). All visual fields were evaluated by the UCSD Visual Field Assessment Center (VisFACT) [25]. Visual fields with more than 33% fixation losses or false-negative errors, or more than 15% false-positive errors, were excluded. The only exception was the inclusion of visual fields with false-negative errors of more than 33% when the field showed advanced disease (worse than −12 dB). SAP tests were defined as normal if the MD and pattern standard deviation was within 95% normal confidence limits and the Glaucoma Hemifield Test (GHT) was also within normal limits. An abnormal SAP test was defined as a visual field with a pattern standard deviation with P<0.05 and/or a GHT outside normal limits.

### Optic Disc Stereophotographs

Simultaneous stereoscopic optic disc photography (Kowa Nonmyd WX3D, software version VK27E, Kowa Company Ltd., Tokyo, Japan) was performed for all subjects and digital stereoscopic images were reviewed with a stereoscopic viewer (Screen-VU stereoscope, PS Mfg., Portland, Oregon, USA) by two or more experienced graders. Each grader was masked to the subject's identity and to the other test results. Details of the methodology employed to grade optic disc photographs at the UCSD Optic Disc Reading Center have been provided elsewhere [24], [26].

### Deep Range Imaging Optical Coherence Tomography

CT was assessed from images acquired using DRI-OCT, which is a new Swept Source OCT, currently not available commercially in the United States. The DRI-OCT acquires 100,000 A-scans per second and provides an axial resolution in tissue of 8 mm. DRI-OCT has a center wavelength of 1,050 nm and a sweeping range of approximately 100 nm, compared to the 850 nm wavelength of SDOCT [23], [27]. For the present study, all eyes were imaged using the wide field 12×9 mm raster scan setting with the scan centered on the posterior pole. It was therefore possible to obtain images of the macular and ONH region in a single scan. The 12×9 mm scan comprises 256 B-scans, each comprising 512 A-scans for a total of 131,072 axial scans/volume. The total acquisition time was 1.3 seconds per 12×9 mm scan.

DRI-OCT segmentation software (version 9.11, Topcon, Inc., Tokyo, Japan) was used to identify the limits of the choroid and determine CT throughout the 12×9 mm scan. Data was exported using the manufacturer's OCT-Batch (version 9.1.10) utility. The quality of each scan and accuracy of the segmentation algorithm were reviewed by an experienced examiner and poor quality images (quality score <50, clipped or poorly focused scans) were excluded.

Scans with segmentation failures and motion artifacts were also excluded.

**Figure 1:**
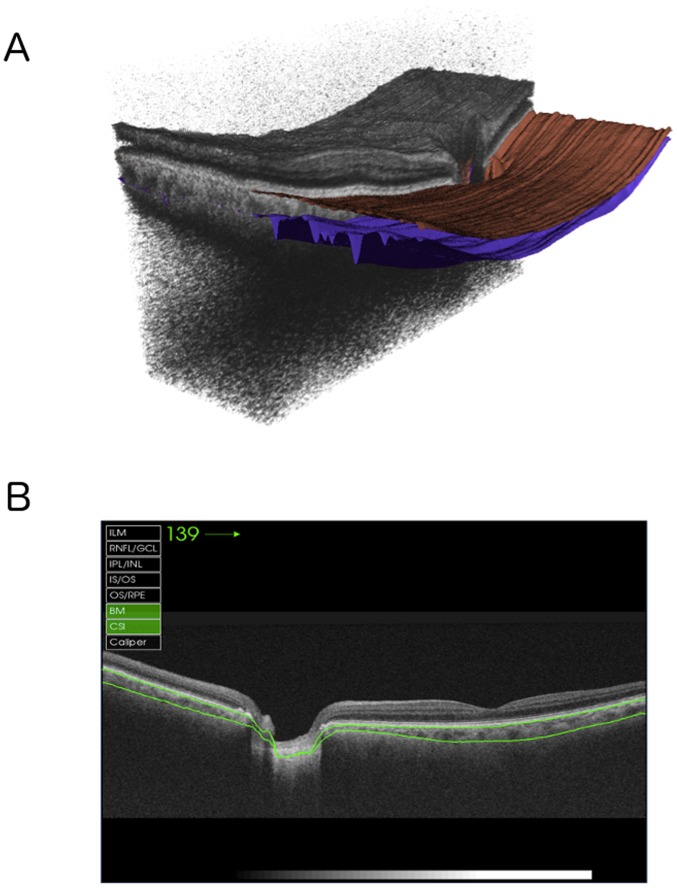
Partial 3-dimensional image of a 12×9 mm scan obtained with DRI-OCT using the instrument's cropping function and showing segmentation of the choroid (A). Horizontal B scan showing a segmented choroid with thickness measured between the two green demarcated lines (B).

**Figure 2:**
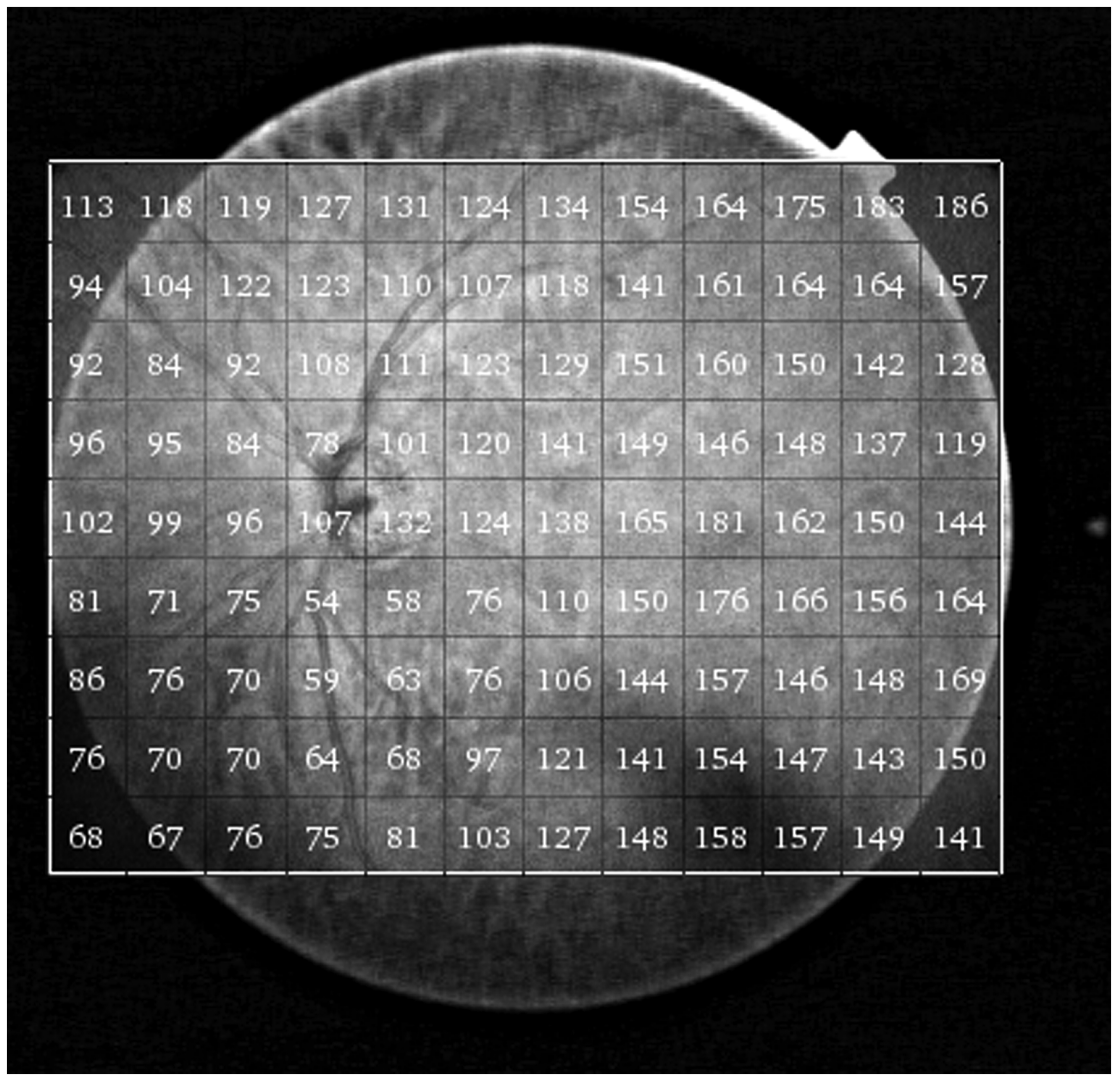
Example of a 12×9 mm DRI-OCT image of a glaucomatous eye included in the study. The numbers correspond to the average choroidal thickness (in µm) for each of the 108 1 mm^2^ regions of the scan.

**Figure 3:**
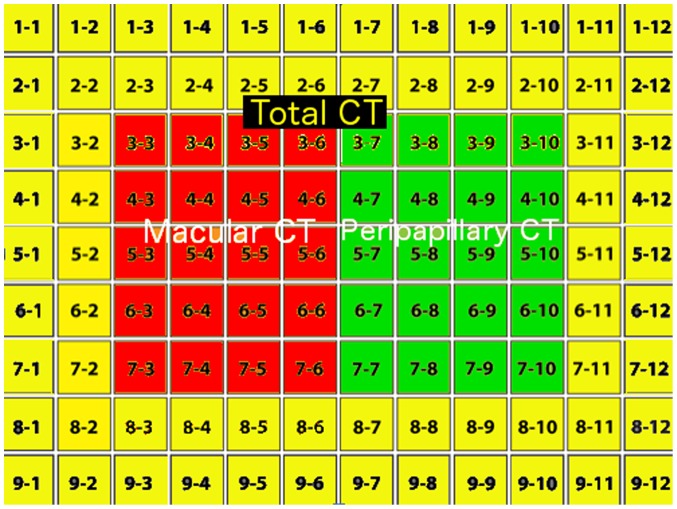
Example of the analysis map for the right eye used in the study. The figure shows the 12×9 mm scan divided into 1 mm^2^ sectors. The areas used for the calculation of macular choroidal thickness (red), peripapillary choroidal thickness (green) and total choroidal thickness (yellow, red and green) are shown. The numbers represent the row and column co-ordinates for each sector.

Figure 1 shows an example of a 3-dimensional SS-OCT scan with the segmented borders of the choroid delineated. The SS-OCT software calculates the average CT for each 1 mm^2^ grid square of the 12×9 mm scan and allows this date to be displayed and exported. Therefore for each eye the average CT in a total of 108 locations was calculated (Figure 2). The mean CT in 20 central 1 mm×1 mm squares was calculated to represent the macular CT (Figure 3). The peripapillary CT was estimated as the mean CT of 20 1 mm×1 mm squares in the region of the optic disc. The total CT was calculated as the mean CT for all sectors in the 12×9 mm^2^ scan. Segmentation software was also used to calculate the average RNFL thickness over the total 12×9 mm^2^ area from the wide field SS-OCT scan. The data for this study is deposited in Table S1.

### Statistical Analysis

Normality assumption was assessed by inspection of histograms and using Shapiro-Wilk tests. Student *t*-tests were used for group comparison for normally distributed variables and Wilcoxon rank-sum test for continuous non-normally distributed variables. The association between CT and parameters including age, axial length, CCT, ocular perfusion pressure, MD, RNFL thickness, gender and ancestry was assessed using scatter plots and univariate linear regression in healthy and glaucomatous eyes. Observations from two eyes of the same subject are likely to be correlated, which can lead to underestimation of true variance. A between-cluster variance estimator was therefore used to account for correlations between eyes of the same subject and calculate robust variance estimates in univariate and multivariable analyses [28]. Variables from univariate analysis with a significance of 0.2 or smaller were examined in multivariable models with total CT as the dependent variable. The analysis was repeated for peripapillary and macular CT. The relationship between CT and glaucoma, and between CT and SAP MD, was examined in the multivariable model accounting for the covariables associated with CT. All statistical analyses were performed with commercially available software (Stata version 13; StataCorp, College Station, TX).

## Results

The study included 265 glaucomatous eyes from 187 patients and 125 healthy eyes from 84 subjects. 10 eyes with poor quality scans (quality score <50 or poorly focused scans) and 58 eyes with scans with segmentation failures and motion artifacts were excluded. This left a total of 216 glaucomatous eyes from 140 patients and 106 healthy eyes from 67 subjects.

**Figure 4:**
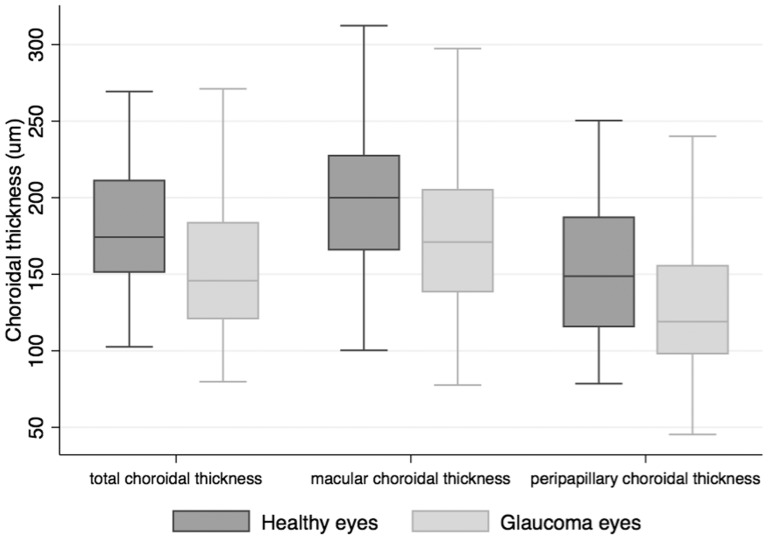
Boxplot illustrating the distribution of total, macular and peripapillary choroidal thickness values in glaucomatous and healthy eyes.

**Figure 5:**
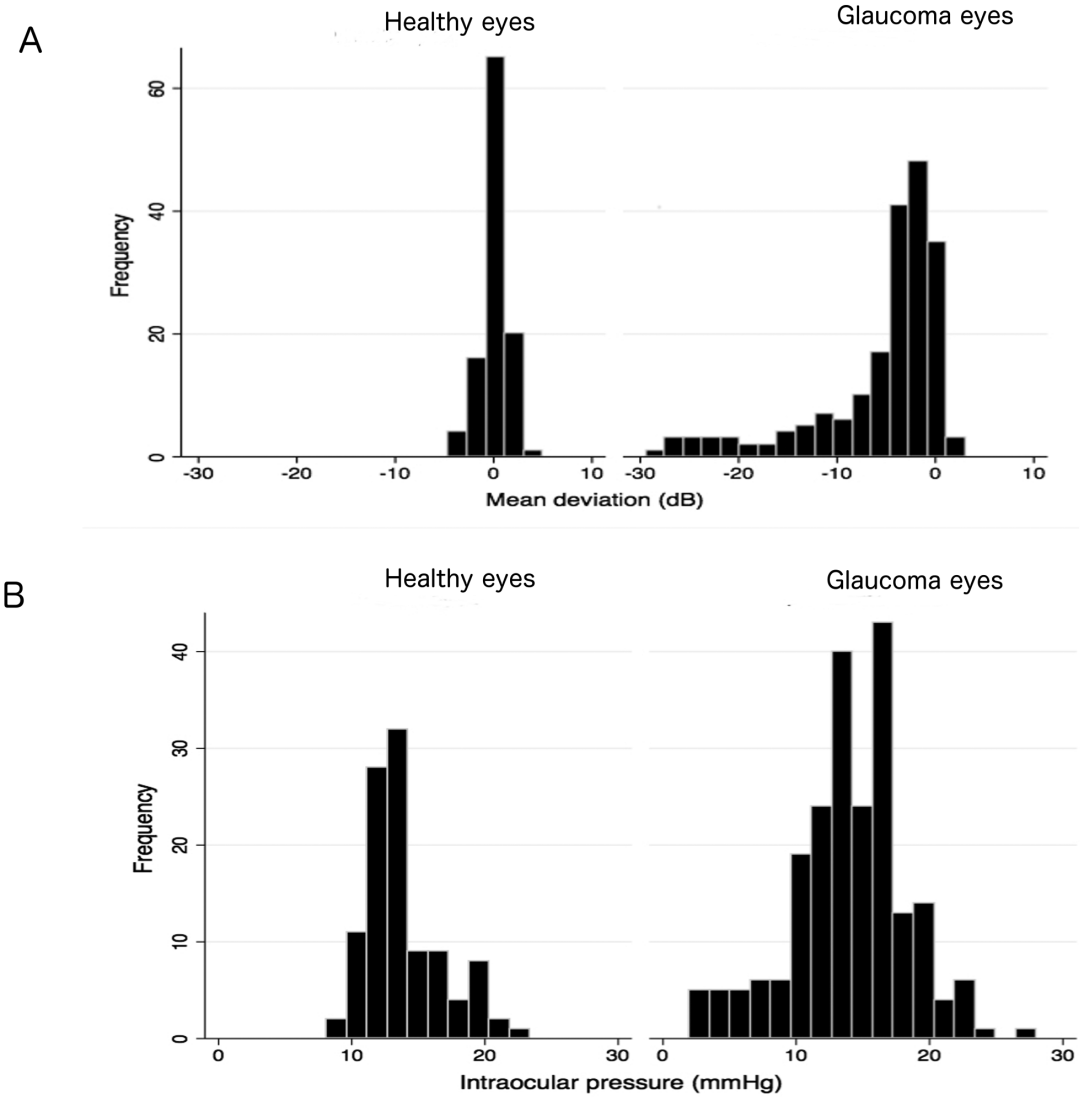
Distribution of standard automated perimetry (SAP) mean deviation (decibels) in healthy and glaucomatous eyes (A). Distribution of intraocular pressure (mmHg) in healthy and glaucomatous eyes (B).

The demographic and clinical characteristics of those included in the study are summarized in Table 1. Subjects with glaucoma were significantly older than healthy subjects (P<0.001). Those with glaucoma had a mean age of 71.8±10.2 years compared to 61.2±11.9 years in healthy subjects. The mean total CT in glaucomatous eyes was 157.7±48.5 µm, which was significantly thinner than in healthy eyes 179.9±36.1 µm (P<0.001) (Table 1 and Figure 4). Peripapillary and macular CT were also significantly thinner in glaucomatous subjects. The mean peripapillary choroidal thickness was 154.1±44.1 µm in healthy subjects compared to only 134.0±56. 9 µm in those with glaucoma (P<0.001). The mean macular CT was 199.3±46.1 µm in healthy subjects compared to only 176.2±57.5 µm in those with glaucoma (P<0.001) (Table 1 and Figure 4). For both glaucomatous and healthy eyes, CT was largest in the macular region. Eyes with glaucoma had longer axial length (P = 0.039), worse SAP MD (P<0.001), thinner RNFL thickness (P<0.001) and higher systolic OPP (P = 0.001) than healthy eyes (Table 1). The distribution of SAP MD and IOP for healthy and glaucomatous eyes is shown in Figures 5A and 5B. Subjects with glaucoma also had higher systolic BP (P<0.001) than healthy subjects. CCT and IOP were not significantly different between groups (P = 0.392 and P = 0.195 respectively).

**Table 1:** Demographic and ocular characteristics of healthy and glaucomatous subjects included in the study.

	Healthy (n = 106 eyes, 67 subjects)	Glaucoma (n = 216 eyes, 140 subjects)	P-value
Age (years)	61.21±11.89	71.82±10.19	<0.001
Sex (female)	69 (65%)	98 (45%)	0.001*
Ancestry			
European	31 (46%)	94 (67%)	0.004 *
African	31 (46%)	33 (24%)	
Other	5 (8%)	13 (9%)	
Axial length (mm)	23.87±1.00	24.13±1.25	0.039
CCT (µm)	547.36 ± 45.21	543.12 ± 39.84	0.392†
Systolic BP (mmHg)	129.15±14.35	136.84±15.96	<0.001
Diastolic BP (mmHg)	77.26±10.33	75.27±9.47	0.183
IOP (mmHg)	13.94±2.79	14.02±4.33	0.195
Systolic OPP (mmHg)	115.21±14.53	122.82±15.90	0.001
Diastolic OPP (mmHg)	63.32±10.20	61.25±9.75	0.140
SAP MD (dB)	0.13±1.30	−5.25±6.29	<0.001
RNFL thickness (µm)	57.37±7.35	41.25±11.23	<0.001
Total CT (µm)	179.90±36.05	157.70±48.54	<0.001
Peripapillary CT (µm)	154.12±44.11	133.99±56.89	<0.001
Macular CT (µm)	199.30±46.10	176.15±57.54	<0.001
CT – inferior (µm)	169.33±38.54	150.96±49.44	<0.001
CT – superior (µm)	190.05±37.59	164.45±51.23	<0.001

Mean ± standard deviation and Wilcoxon rank sum test unless specified otherwise. *Fisher's exact test.

† Student t-test. Abbreviations: CCT  =  Central corneal thickness; BP  =  blood pressure; IOP =  intraocular pressure; OPP  =  ocular perfusion pressure; SAP  =  standard automated perimetry; MD  =  mean deviation; RNFL  =  retinal nerve fiber layer; CT  =  choroidal thickness.

Where appropriate P-values were adjusted for correlations between eyes of the same subject using a between-cluster variance estimator.

**Figure 6:**
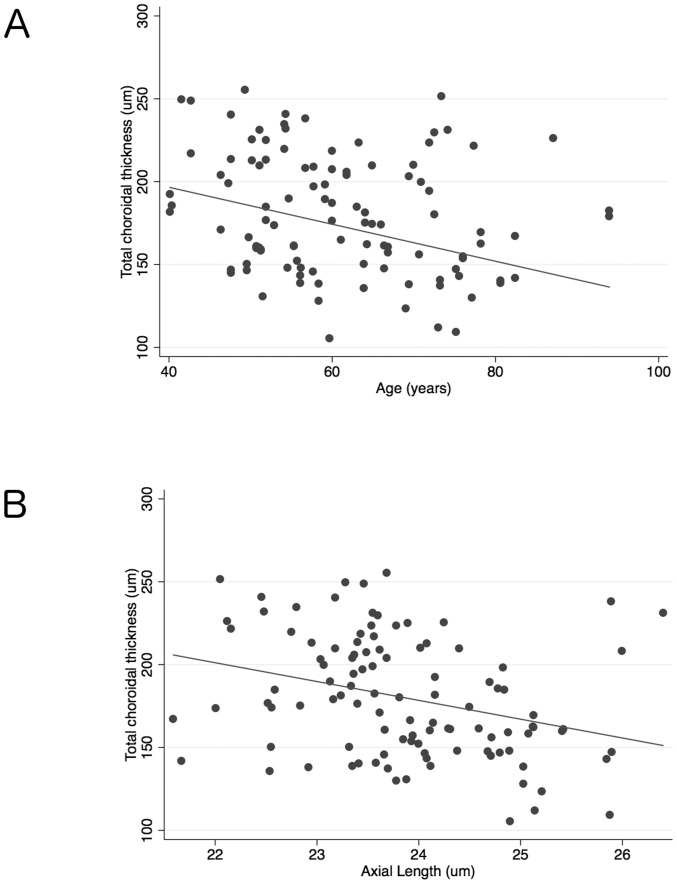
Scatter plots showing the relationship between total choroidal thickness and age in healthy eyes (R^2^ = 0.057, Slope: −0.72 µm/year, P = 0.039) (A) and the relationship between axial length and total choroidal thickness in healthy eyes (R^2^ = 0.099, Slope: −11.36 µm/year P = 0.027) (B).

The relationship between total CT and variables including age, gender, ancestry, axial length, systolic and diastolic OPP, and CCT was examined in healthy subjects. Scatter plots suggested a tendency for total CT to be lower in older subjects and those with longer axial length (Figures 6A and 6B). Univariable linear regression analyses also indicated longer axial length (P = 0.027) and older age (P = 0.039) to be associated with thinner total CT in healthy eyes (Table 2). Other variables including gender, ancestry, OPP and CCT were not significant. In the multivariable analysis, axial length (P = 0.008) and age (P = 0.007) remained significantly associated with total CT in healthy subjects. Each decade of increasing age was associated with a 8.6 µm decrease in total CT and each 1 mm longer axial length, a 12.7 µm decrease in total CT in healthy eyes (Table 3). In addition, age was significantly associated with peripapillary CT (P = 0.038), and axial length was significantly associated with peripapillary CT (P = 0.001) in healthy eyes. In contrast, macular CT was not significantly associated with age (P = 0.064) and axial length (P = 0.078) in healthy eyes in the multivariable models (Table 3).

**Table 2:** Results of the univariable regression analyses evaluating the association between choroidal thickness and clinical variables in healthy eyes.

	Total Choroidal Thickness		Peripapillary Choroidal Thickness		Macular Choroidal Thickness	
	Coefficient (95% CI)	P- value	Coefficient (95% CI)	P-value	Coefficient (95% CI)	P-value
Age (years)	−0.72	0.039	−0.72	0.14	−0.67	0.124
	(−1.41 to 0.04)		(−1.66 to 0.23)		(−1.52 to 0.19)	
Gender	4.35	0.620	8.37	0.457	6.25	0.537
	(−13.08 to 21.78)		(−13.99 to 30.72)		(−13.85 to 26.35)	
Ancestry	0.99	0.911	9.54	0.381	−16.23	0.136
	(−16.68 to 18.66)		(−12.04 to 31.11)		(−37.71 to 5.25)	
Axial length (mm)	−11.36	0.027	−15.01	0.007	−8.96	0.133
	(−21.38 to −1.35)		(−25.74 to −4.27)		(−20.71 to 2.80)	
Systolic OPP (mmHg)	−0.248	0.441	−0.24	0.546	−0.47	0.129
	(−0.89 to 0.39)		(−1.04 to 0.56)		(−1.08 to 0.14)	
Diastolic OPP (mmHg)	−0.31	0.478	−0.21	0.704	−0.80	0.105
	(−1.17 to 0.55)		(−1.33 to 0.90)		(−1.77 to 0.17)	
CCT (µm)	0.03	0.770	−0.02	0.865	0.04	0.664
	(−0.15 to 0.21)		(−0.25 to 0.21)		(−0.16 to 0.24)	
Systolic BP (mmHg)	−0.37	0.245	−0.26	0.509	−0.509	0.147
	(−1.01 to 0.26)		(−1.05 to 0.53)		(−1.20 to 0.18)	
Diastolic BP (mmHg)	−0.48	0.256	−0.18	0.730	−0.81	0.08
	(−1.31 to 0.35)		(−1.20 to 0.85)		(−1.73 to −0.11)	
IOP (mmHg)	−1.00	0.512	0.50	0.791	−0.58	0.761
	(−4.04 to 2.03)		(−3.26 to 4.27)		(−4.36 to 3.20)	

Abbreviations: OPP  =  ocular perfusion pressure; CCT  =  central corneal thickness; BP  =  blood pressure; IOP =  intraocular pressure.

**Table 3:** Results of multivariable analysis for total, macular and peripapillary choroidal thickness in healthy eyes including variables from univariable analysis with P<0.2.

Characteristic	Coefficient	95% CI	P-value
**Total CT**			
Constant (µm)	537.24	314.75 to 759.74	<0.001
Age (per decade older)	−8.62	−14.76 to −2.48	0.007
Axial length (per 1 mm longer)	−12.70	−21.91 to −3.49	0.008
**Peripapillary CT**			
Constant (µm)	600.23	364.16 to 836.30	<0.001
Age (per decade older)	−8.96	−17.41 to −0.50	0.038
Axial length (per 1 mm longer)	−16.39	−26.26 to −6.52	0.001
**Macular CT**			
Constant (µm)	553.95	284.00 to 823.89	<0.001
Age (per decade older)	−7.76	−15.98 to −0.46	0.064
Axial length (per 1 mm longer)	−10.16	−21.50 to 1.18	0.078

Abbreviations: CT  =  choroidal thickness.

A similar analysis was performed using glaucomatous and healthy eyes and for macular and peripapillary CT. Age and axial length were associated with thinner total, peripapillary and macular CT (Table 4). In univariable models thinner RNFL thickness was also associated with thinner total, peripapillary and macular CT (P<0.001 for all comparisons) (Table 4). Variables with P<0.2 in the univariable analyses were examined using multivariable models (Table 5). Age and axial length remained significantly associated with total, peripapillary and macular CT. Once age and axial length were corrected for, the presence or absence of glaucoma had no significant relationship on total (P = 0.216), peripapillary (P = 0.417) or macular CT (P = 0.330) (Table 5). Similar multivariable models were constructed to examine the relationship between RNFL thickness, age, axial length and CT. RNFL thickness was not significant in the total CT (coefficient  = 0.03, 95% CI: −0.01 to 0.06, P = 0.107) analyses.

**Table 4:** Results of univariable regression analyses evaluating the association between choroidal thickness (total, peripapillary and macular) and demographic and clinical characteristics in healthy and glaucomatous eyes.

	Total Choroidal Thickness		Peripapillary Choroidal Thickness		Macular Choroidal Thickness	
	Coefficient (95% CI)	P- Value	Coefficient (95% CI)	P-value	Coefficient (95% CI)	P-value
**Age (years)**	−1.12	<0.001	−0.98	0.003	−1.21	<0.001
	(−1.66 to −0.57)		(−1.61 to −0.35)		(−1.87 to −0.54)	
**Gender**	−14.76	0.021	−13.33	0.071	−12.82	0.071
	(−27.27 to −2.24)		(−27.83 to 1.17)		(−27.79 to 2.16)	
**Ancestry**	12.05	0.081	18.72	0.02	0.23	0.976
	(−1.52 to 25.63)		(2.94 to 34.50)		(−15.47 to 15.00)	
**Axial length (mm)**	−12.25	<0.001	−14.33	<0.001	−12.34	<0.001
	(−17.51 to −6.99)		(−20.27 to 8.40)		(−18.36 to −6.32)	
**Systolic OPP (mmHg)**	−0.23	0.259	−0.21	0.349	−0.16	0.493
	(−0.63 to 0.17)		(−0.66 to 0.24)		(−0.61 to 0.30)	
**Diastolic OPP (mmHg)**	0.39	0.188	0.24	0.493	0.37	0.302
	(−0.19 to 0.96)		(−0.44 to 0.92)		(−0.34 to 1.08)	
**CCT (µm)**	0	0.956	−0.04	0.666	0.02	0.865
	(−0.15 to 0.15)		(−0.21 to 0.14)		(−0.16 to 0.19)	
**RNFL thickness (µm)**	1.10	<0.001	1.19	<0.001	1.14	<0.001
	(0.63 to 1.56)		(0.65 to 1.71)		(0.58 to 1.70)	
**SAP MD (dB)**	0.62	0.402	0.49	0.543	0.81	0.366
	(−0.83 to 2.06)		(−1.09 to 2.06)		(−0.95 to 2.57)	
**Glaucoma**	−22.20	<0.001	−20.14	0.005	−23.15	0.001
	(−33.74 to −10.65)		(−34.01 to −6.26)		(−37.15 to −9.14)	

Abbreviations: OPP  =  ocular perfusion pressure; CCT  =  Central corneal thickness; RNFL  =  retinal nerve fiber layer; SAP  =  standard automated perimetry; MD  =  mean deviation.

**Table 5:** Results of multivariable analysis including variables from univariable analysis with P<0.2 for healthy and glaucomatous eyes.

Characteristic	Coefficient	95% CI	P-value
**Total CT**			
Constant (µm)	548.70	428.38 to 669.02	<0.001
Age (per decade older)	−10.78	−16.19 to −5.34	<0.001
Axial length (mm)	−12.69	−17.64 to −7.73	<0.001
Glaucoma (yes)	−7.45	−19.29 to 4.38	0.216
**Peripapillary CT**			
Constant (µm)	566.52	414.95 to 690.83	<0.001
Age (per decade older)	−9.80	−1.61 to −0.35	0.003
Axial length (mm)	−14.76	−20.54 to −9.00	<0.001
Glaucoma (yes)	−5.89	−20.16 to 8.36	0.417
**Macular CT**			
Constant (µm)	577.86	436.63 to 719.08	<0.001
Age (per decade older)	−11.74	−18.52 to −4.96	0.001
Axial length (mm)	−12.85	−18.56 to −7.14	<0.001
Glaucoma (yes)	−7.33	−22.14 to 7.47	0.330

Abbreviations: CT =  choroidal thickness.

## Discussion

In the present study SS-OCT was used to measure CT in healthy and glaucomatous eyes. The wide-field SS-OCT scan allowed imaging of the macular and peripapillary regions in a single scan, and over a wider area than previously studied. Although we found an association between glaucoma and thinner choroid in the univariable analysis, any association disappeared after adjusting for differences in age and axial length between groups. Older age and longer axial length were both associated with thinner choroid, suggesting that the apparent association between glaucoma and thinner choroid was in fact due to the confounding effect of age and axial length. Accounting for age and axial length, there was also no significant relationship between glaucoma and thickness of the macular or peripapillary choroid.

We also examined the relationship between CT and markers of glaucoma severity, however there was no significant association between SAP MD and total, macular or papillary CT. Thinner RNFL was associated with thinner choroid in univariable analyses (Table 4), however, RNFL thickness was not significant in the multivariable analyses, accounting for age and axial length. The likely explanation for this is that, similarly to choroidal thickness, RNFL thickness decreases with age and is also thinner in eyes with large axial length [29]–[32].

These findings are in agreement with previous studies using EDI-OCT suggesting that in vivo measurements of CT are not significantly different in patients with open angle glaucoma compared to controls [15], [16], [18], [33], [34]. However, our study goes further as most previous studies have only examined the thickness of the choroid in the macular region and have not used SS-OCT technology or employed a wide-field scanning technique to image a greater choroidal area.

Due to the proximity of the peripapillary choroid to the ONH, one might suppose that peripapillary CT would be a better measure of the blood supply to the ONH and therefore of more relevance to glaucoma. There is some evidence that peripapillary CT might be important in specific subtypes of glaucoma [35]–[36]. For example, Roberts and colleagues found peripapillary CT was 25 to 30% thinner in those with sclerotic glaucomatous disc damage than in patients with focal and diffuse optic disc damage or healthy controls [35]. However several other studies have not detected an association between glaucoma and peripapillary CT [9], [37], [38]. In a study of eyes with glaucoma without history of raised IOP, Hirooka and colleagues suggested that the portion of the macular choroid closest to the ONH may be thinner in those with glaucoma [35]. There was also correlation between CT in this region and SAP MD. To investigate this possibility in our sample, we calculated CT in the zone between the macula and ONH (measured as the average CT in the 20 squares in region 3–5 to 3–8×7–5 to 7–8 in Figure 3) but found no association between CT and glaucoma or between CT in this region and SAP MD. However, our sample was not selected to include only eyes with normal intraocular pressure.

The current study has provided useful information about the normal choroid. The results indicate that CT is largest at the macula and decreases towards the peripapillary region. The macular choroid was typically 29% thicker than the peripapillary choroid in healthy subjects. The finding that CT is greatest near the macula is consistent with previous studies, however, overall the CT measurements in this study were slightly thinner than those previously reported using both SS-OCT and EDI-OCT [14], [20], [21], [39]. For example, two recent studies, which used SS-OCT to examine CT in healthy subjects, found average subfoveal CT to be approximately 270 to 280 µm. In comparison, using EDI-OCT subfoveal CT using SD-OCT was 263 µm to 273 µm [40], [41]. These results suggest that EDI-OCT and SS-OCT produce similar CT measurements. The thinner choroid noticed in the present study is likely to be due to differences in patient characteristics between the studies. Another possible explanation is that previous studies relied on manual segmentation and measured a few points along the choroid rather than the large area measured in the present study.

Previous studies have shown an association between thinner choroid and older age, longer axial length, thicker CCT, and lower ocular perfusion pressure [13], [15], [21], [42]–[44]. We found thinner CT was associated with longer axial length and older age, however, there was no significant relationship between CT and gender, ancestry, systolic or diastolic OPP, or CCT. In healthy eyes total CT was inversely proportional to age with an approximate 9 µm decrease in CT with each decade. This is slightly smaller than the 16 to 31 µm per decade decrease in macular CT previously reported in studies using EDI-OCT but could be explained by the thinner overall CT in the present study [15], [16], [20], [29], [45]. In multivariable models, accounting for axial length, we found a similar relationship between age and peripapillary CT (9.0 µm decrease per decade) and macular CT (7.8 µm decrease per decade) (Table 3). In healthy eyes the total CT also decreased by almost 13 µm for each 1 mm increase in axial length, compared to a decrease in peripapillary CT of 16.4 µm, and a decrease in macular CT of 10.2 µm, for similar increases in axial length. Similar results were found in the analyses including all eyes (Table 3).

Interestingly, we found the regression coefficient between age and choroidal thickness was smaller in healthy subjects compared to those with glaucoma (−0.72 (95% CI = −1.41 to 0.04) versus −1.03 (95% CI = −1.65 to −0.40). This indicates that the relationship between older age and thinner choroid may be steeper in glaucomatous eyes than controls, or in other words, there may be a greater decrease in CT with aging in those with glaucoma compared to controls. Although the confidence intervals overlap, indicating the differences in slopes of change in CT did not reach statistical significance; it raises the possibility that there may be an interaction between glaucoma, aging and changes in choroidal thickness over time. It would be interesting to evaluate this possible interaction in a longitudinal study, using age-matched glaucomatous and healthy subjects.

There were limitations to the present study. 68 of 390 eyes (17.4%) were excluded due to poor scan quality or segmentation failures, which potentially could have introduced bias. For example, it is possible that eyes with thicker choroid may have been more difficult to image due to deeper penetration needed. However, it was important to have a rigorous quality control to ensure included scans were accurate and significant bias seems unlikely as the proportion of glaucomatous and healthy eyes excluded was similar (49 of 265 (18.5%) versus 19 of 125 (15.2%) respectively). A further potential limitation of this study was that patients with glaucoma were already receiving treatment, For this reason, the study had limited ability to evaluate the association between IOP and CT and IOPs as shown from the observation that IOPs were not significantly different between glaucomatous and healthy eyes (Table 1 and Figure 5B). It is possible that CT may be different in untreated subjects, with higher IOPs. Using CT measurements obtained with radiofrequency, Cristini and colleagues found glaucomatous eyes with elevated IOP (30 to 45 mmHg) had 20% greater CT than healthy eyes [46]. Using EDI-OCT, Usui and colleagues found increased CT with IOP reduction following trabeculectomy [47]. In contrast, it has also been observed that in eyes with acute primary angle closure, a reduction in CT may occur following successful reduction in IOP [48]. Choroidal thinning has also been found to accompany acute increases in IOP induced by darkroom prone provocation testing in those with suspected primary angle closure [50]. It was proposed that raised IOP might induce choroidal thinning secondary to an IOP-induced reduction in choroidal blood flow [51], [52]. A further limitation of our study is we did not regulate patients fluid intake prior to testing. This is a potential problem as hydration status is likely to influence CT, with an increase in CT reported after water drinking. In future studies it may be important to regulate subjects' fluid intake prior to imaging [49]. We did not investigate choroidal blood flow. Although the majority of total ocular blood volume and flow (∼80–90%) is derived from the choroidal vascular, a causal relationship between measurements of ocular blood flow and glaucoma progression has not been established [53]. In addition, we estimated the macular and peripapillary CT using the grid pattern provided by the SS-OCT software which was centered on the fovea. Although during scanning the SS-OCT device uses a fixation target to center the grid on the fovea, it is possible that the macular and peripapillary CT measurements may not have been obtained at the same location relative to the macula and optic disc in every patient. For this reason we reviewed each scan and included only those passing the quality control process described in the methods.

In conclusion, this study has shown that SS-OCT is a useful tool for evaluation of CT. Using cross-sectional data we have demonstrated a relationship between increasing age, longer axial length and thinner choroid. However, when differences in age and axial length between glaucomatous and healthy subjects were accounted for there was no association between glaucoma and CT. Although we found no relationship between glaucoma and CT, prior studies have indicated an association between glaucoma and both impaired choroidal circulation and decreased blood flow to the ONH [52], [54]–[55]. It may be that CT is a poor marker of functional integrity of the choroid, particularly as the choroid has an extravascular space that could explain much thickness variability. Further studies that explore the relationship between CT and choroidal blood flow may provide further insight into the potential role of the choroid in glaucoma.

## Supporting Information

Table S1 **Choroidal thickness dataset.** The dataset contains data for all 216 glaucomatous eyes and 106 healthy eyes included in the study.
(CSV)
